# Assessing early child development and its association with stunting and schistosome infections in rural Zimbabwean children using the Griffiths Scales of Child Development

**DOI:** 10.1371/journal.pntd.0009660

**Published:** 2021-08-11

**Authors:** Francisca Mutapi, Lorraine Pfavayi, Derick Osakunor, Rivka Lim, Maritha Kasambala, Arnold Mutemeri, Simbarashe Rusakaniko, Dixon Chibanda, Takafira Mduluza

**Affiliations:** 1 Institute of Immunology & Infection Research, University of Edinburgh, Ashworth Laboratories, Edinburgh, United Kingdom; 2 NIHR Global Health Research Unit Tackling Infections to Benefit Africa (TIBA), University of Edinburgh, Ashworth Laboratories, Edinburgh, United Kingdom; 3 Nuffield Department of Medicine, Centre for Tropical Medicine and Global Health, University of Oxford, Oxford, United Kingdom; 4 School of Life Sciences, University of KwaZulu-Natal; Westville, South Africa; 5 NIHR Global Health Research Unit Tackling Infections to Benefit Africa (TIBA) Zimbabwe, Faculty of Medicine and Health Sciences, University of Zimbabwe, Harare, Zimbabwe of Zimbabwe; 6 Faculty of Medicine and Health Sciences, University of Zimbabwe, Parirenyatwa Hospital, Harare, Zimbabwe; 7 Department of Psychiatry, College of Health Sciences, University of Zimbabwe, Parirenyatwa Hospital, Harare, Zimbabwe; 8 Department of Biochemistry, University of Zimbabwe, Harare, Zimbabwe; NIPD: National Institute of Parasitic Diseases, CHINA

## Abstract

There is a paucity of reference early childhood development (ECD) data at community level in rural Africa. Our objective was to conduct a comprehensive assessment of ECD in rural Zimbabwe and determine the impact of stunting and schistosome infections on ECD. Using the Griffiths Scales of Child Development, we conducted a cross sectional assessment of Eye and Hand Coordination (EHC), Personal-Social-Emotional (PSE), Language and Communication (LC), Foundations of Learning (FL) and Gross Motor (GM) domains and the summary General Development (GD) in 166 children aged 6–72 months. The effects of stunting, malnutrition and *Schistosoma haematobium* infection on ECD was determined. The impact of praziquantel curative treatment of schistosome infection on the developmental scores was determined through a longitudinal follow up at 6 and 12 months. From an initial 166 children, 11 were found to have developmental deficits warranting further investigation. Of the remaining 155, 58.7% recorded a good (≥ average) score for the overall General Development (GD). Proportions of children scoring above the cut-off (≥ average) for each domain were GM (84.5%), PSE (80.6%), EHC (61.9%), FL (43.9%) and LC (44.5%). The prevalence of stunting was 26.8% (95% CI = 20.1%–34.8%) Scores for stunted children were significantly lower for EHC (p = 0.0042), GM (p = 0.0099), and GD (p = 0.0014) with the fraction of lower scores attributable to stunting being GM = 63.4%, GD = 46.6%, EHC = 45%, and LC = 21%. *S*. *haematobium* infection prevalence was 39.7% and mean infection intensity was 5.4 eggs/10 ml urine. Infected children had poorer cognitive performance scores for the FL (p = 0.0005) with 30.8% of poor FL attributable to the infection. Performance in all domains improved to the expected normal or above reference levels at 6 and 12 months post curative treatment of schistosome infections. Our study documented reference values for ECD in rural Zimbabwean children. The study detected deficiencies in the FL domain, which were more pronounced in children, infected with schistosomes, highlighting the need for provision of cognitive stimulation tools and access to early childhood foundation education. There is also need for improved child nutrition and treatment of schistosome infections to improve child development outcomes.

## Author summary

There is a paucity of comprehensive early childhood development data at community level in rural Africa. We assessed the development of rural Zimbabwean children aged 6–72 months and determined the impact of stunting and schistosome infections on their 5 developmental domains; Eye and Hand Coordination, Personal-Social-Emotional, Language and Communication, Foundations of Learning and Gross Motor domains and the summary General Development. We have demonstrated that just over 50% of Zimbabwean children in rural areas are on course for normal child development; with many being more advanced for their age in Gross Motor and Personal-Social-Emotional domains. We also demonstrate that the children face developmental challenges in early childhood, particularly in Foundations for Learning which represents critical psychometric constructs and cognitive skills for learning, executive function, ways of thinking, problem solving, organizing and information planning, analytic thought and memory. We further demonstrate that the poor development scores especially in Foundations for Learning were also attributable to stunting and schistosome infection, with the impact of the latter being reversed by curative antihelminthic treatment. Taken together, the findings strengthen the call for the treatment of paediatric schistosomiasis, accessibility to cognitive stimulation tools and improved nutrition to improve childhood health outcomes.

## Introduction

The early childhood period is considered the most important developmental phase throughout the lifespan of a child with events occurring in these first few years being critical for the child’s developmental trajectory and life course. https://www.who.int/social_determinants/themes/earlychilddevelopment/en/). These events influence the child’s mental and physical health, education and future economic participation.

In most African countries, child development in rural areas is monitored as part of the monthly growth monitoring in child health programs. However, these programs mostly consist of weighing, height measuring, and nutrition advice to caregivers. In Zimbabwe, the growth-monitoring program is part of the child health surveillance system, which uses basic measures such as weight-for-age, height-for-age and mid-upper arm circumference (MUAC) to chart child development. This is conducted at primary health centres with input from village health workers who monitor the number of eligible children in their communities. Nonetheless, a study in Mutasa District in Zimbabwe in 2015 indicated that 70% of children below 5 years of age missed their monthly weighing, meaning that for most children, subtle changes and malnutrition went undetected [1].

In addition to challenges arising from the low uptake of growth monitoring appointments, there is little provision of tools to detect cognitive development. This is due to a shortage of a trained workforce and tool kits for the direct assessment of child development. Community level data is often obtained using screening tools. Screening is conducted via interview of caregivers or through observation of limited sets of actions/behaviours representing a domain of development; they are reliant on predetermined cut-off points to identify children requiring a comprehensive assessment. Tools for comprehensive child development assessment such as the Griffiths Assessment Tool, the Cognitive Adaptive Test/Clinical Linguistic, and Auditory Milestone Scale (CAT/CLAMS) and Ages and Stages Questionnaire (ASQ) that have been assessed in South Africa [2] require trained personal and the assessment of each child is time-consuming. Thus, there is a paucity of comprehensive early childhood development (ECD) data at community level.

Our first aim was to conduct a comprehensive assessment of ECD in rural Zimbabwe to provide, for the first time, data on delays and changes that may have a significant impact on subsequent development in the children. The latest UNICEF Country Profiles for Early Childhood Development report (https://nurturing-care.org/resources/country-profiles/) indicates that 46% of Zimbabwe’s children are at risk of poor development. Therefore, there is a need to identify these children and the causes of their poor development to implement context-specific interventions.

There is also a need to identify local factors contributing to poor child development. A recent analysis of indicators of early childhood experiences and outcomes in low and middle-income countries comprised of 135 national household surveys, collected between 2010 and 2018 from 94 countries indicated that children in sub-Saharan Africa were exposed to stunting or extreme poverty [3]. The study also highlighted that the children had less home stimulation and low attendance at early childcare education; indicators associated with a poorer status in subsequent school learning, labour market productivity and health [3]. Furthermore, the analysis also showed that children in urban areas or those in the richest household scored better on all indicators of child development than those in rural areas or those from poorer households. The authors of this study correctly highlight that these studies were handicapped by the lack of reliable data from several low-income countries and in cases where there were data available; there was poor representation of rural communities [3]. Our current study added data to the poorly represented rural populations.

The detrimental impact of helminth infections on childhood development has been highlighted in previous studies. In a study in Peru, infection with soil-transmitted helminths (STH) has been shown to reduce verbal IQ scores [4]. Furthermore, a meta-analysis of 36 studies of 12,920 children [5] indicated that STH infection compromised learning, memory and intelligence [5]. This study also demonstrated that children successfully treated for the STH infections scored better in these domains than untreated children. Quantitative studies have suggested that schistosome infections are associated with stunting and undernutrition and that both these effects can be reversed through antihelminthic treatment [6], while meta-analysis has indicated that schistosome infections were significantly associated with educational, learning, and memory deficits in school-aged children [7]. However, to date, there has been no longitudinal study investigating the impact of schistosome infection on ECD, focusing on children aged 6 years and below. Therefore, our second aim was to determine if *Schistosoma haematobium* infection compromised any of the child development domains. We further determined if the curative antihelminthic effect improved the performance of previously infected children as determined by their performance in child development assessments.

## Methods

### Ethical approval and consent

This study was part of a larger study investigating the overall health impact of paediatric schistosomiasis in children aged 6 years and below, from 2017 to 2019. The study received institutional approval from the University of Edinburgh and Ethical approval from the Medical Research Council of Zimbabwe (MRCZ/A/2246 and MRCZ/A/2573). Permission to conduct the study in the province was obtained from the Provincial Medical Director. Prior to enrolment, the study aims, and procedures were explained to all participants and their parents/guardians in English or the local language, Shona. Written informed consent was obtained from the participants’ parents/guardians as appropriate. Recruitment into the study was voluntary and parents/guardians were free to withdraw the participants at any time with no further obligation.

### Study area

The study was conducted in Murewa district located in Mashonaland East province of Zimbabwe (17°38′49″S 31°46′39″E). Murewa District is one of seven districts in the Mashonaland East province of Zimbabwe, whose people are primarily subsistence farmers producing maize and vegetables which form the stable diet. This is an area we have previously worked in and reported on water contact and usage [8]. The area was selected for this study because the prevalence of *S*. *haematobium* is high (>50%) while the prevalence of *S*. *mansoni* and soil transmitted helminths (STH) is low (<15%) [9]. As we have previously reported [10], this area was included in Zimbabwe’s helminth control program running annually from September 2012 to November 2017 where schistosomiasis was treated via mass drug administration of praziquantel to all school children. In this area there was mopping up treatment exercise in January—February 2018. During the MDA only school children aged 6 years and above are treated, meaning that preschool children are excluded from MDA, a health inequality we have previously highlighted [11]. Since 2018, there has not been an MDA exercise in this area and the children recruited in this study had all never been treated for schistosomiasis.

### Study design

The study comprised of a cross sectional study design followed by a 1-year longitudinal study (see S1 Fig). The longitudinal study had an intervention aspect; schistosome-infected children were treated with praziquantel and followed up at 6 weeks to check treatment efficacy. Children were then followed up at 6 months and 12 months to determine the impact of treatment on their performances in the child development assessments. Children were recruited from crèches and early child development centres. Parents/guardians of children not attending any of the educational programmes brought the children to meeting points used by the community for the Expanded Program for Immunisation (e.g., local school, or primary health centre) for enrolment into the project. A questionnaire designed in English and translated into the local dialect (Shona) was administered to gather demographic data and establish exposure behaviour to schistosome infective freshwater sources.

### Study inclusion and exclusion criteria and study population

At baseline, the study enrolled children aged 6 months to 72 months who met the following inclusion criteria. The children had to, i) be lifelong residents of the study area, ii) not have previously received antihelminthic treatment, iii) be negative for *S*. *mansoni* and STH (in practical terms, as the prevalence of these is very low in the area, no children were excluded on this criteria), and iv) guardian/career had given consent for them to participate in the study. Following these inclusion criteria, 166 children were included in the cross-sectional study.

To be included in the longitudinal cohort, children who fulfilled the inclusion criteria described above had to meet the additional criteria of; v) having full ECD assessment and; vi) not be suffering from any severe developmental disorder as diagnosed from the baseline assessments. Children with Development Quotient (DQ) scores below 50, were excluded from the longitudinal study as this score and below indicate a level of development impairment [12] requiring further clinical investigation or intervention. Following these inclusion criteria, 79 children were included in the longitudinal study.

### Anthropometry

Weight (nearest 0.1 kg) and height (nearest 0.1 cm) without shoes and in light clothing were measured using an electronic scale and a stadiometer respectively. For babies, height was measured with an infantometer baby board, and weight with a baby scale. The Mid-Upper Arm Circumference (MUAC) was measured (nearest 1mm) using a child MUAC tape on the left arm, midpoint between the shoulder and the tip of the elbow, with the arm relaxed and hanging down the body. Growth and nutritional status were assessed using the WHO Anthro software, version 3.0.1 (http://www.who.int/childgrowth/en/) [13]. This generated Z- scores for specific measures of nutrition and growth, i.e. stunting by Height-for-Age (HAZ), underweight by Weight-for-Age (WAZ) and BMI-for-Age (BAZ), and malnutrition by MUAC and Weight-for-Height (WHZ). Measures were considered abnormal when Z scores were < -2 Z–scores [14].

### Parasitological diagnosis

A sample of urine, about 50ml volume, was collected from each participant on three consecutive days and a stool specimen was collected on a single day from each participant. Samples were collected between 10:00h and 14:00h and processed within 2 hours of collection. Urine samples were examined microscopically for *S*. *haematobium* infection following the standard urine filtration method [15] and the number of eggs was reported per 10 ml of urine. Stool samples were processed using the Kato–Katz method [16] and parasite eggs also enumerated under light microscope for *S*. *mansoni* (in duplicates) and results reported per gram of stool.

Children were diagnosed positive for helminth infection if at least one parasite egg was detected in their urine or stool samples. All children who were positive for *S*. *haematobium* infection were treated with a single dose of praziquantel at the standard 40 mg/kg body weight after their early child development assessments. Tablets were crushed and administered with squash and sliced bread [17] by local nurses. A post-treatment efficacy check (egg count) was carried out for all treated participants at 6 weeks post treatment.

### Developmental assessment

The child development assessment was conducted using the Griffiths Mental Developmental Scales III for children aged 72 months (6 years) and below [12]. Children were assessed at baseline and at 6 and 12 months post-treatment. The Griffiths Mental Developmental Scales III tool was selected, as it is comprehensive in assessing all developmental domains. The domains measured by this tool are Eye and Hand Coordination, (EHC), Personal-Social-Emotional (PSE), Language and Communication (LC), Foundations of Learning (FL), Gross Motor Function (GM). This assessment gives standardized sub-quotient scores for each domain. The Griffiths Mental Developmental Scales allow for the calculation of a summary General Development (GD) quotient which is derived using each of the measures for the 5 individual domains. It is an indicator of a child’s growth and development across the range of psychosocial competencies. This tool has been validated in South Africa [18] and prior to our study, we validated the tool to ensure relevant contextual language and phrases were used during the assessment. Clinical psychologists who had all completed an accredited training course on the Griffiths Mental Developmental Scales III (led by AM) assessed the children. Data were captured electronically and underwent quality control checks on the day.

### Data handling and statistical analysis

Growth and nutritional indices adjusted for age and expressed as Z-scores [13], were calculated using the WHO Anthro software, version 3.0.1 for children ≤60 months (http://www.who.int/childgrowth/en/) and the WHO Anthro plus software for children ≥60 months (https://www.who.int/growthref/tools/en/). Based on raw anthropometric (including weight and height) and demographic data (including age and sex), anthropometric estimates based on length/height–for–age, weight–for–age, and body mass index (BMI)–for–age were used to generate Z-scores from which growth and nutritional status was measured. Stunting was determined by height–for–age Z-scores (HAZ), and malnutrition by weight–for–age Z-scores (WAZ) and BMI–for–age Z-scores (BAZ). Measures with Z-scores < -2 were considered abnormal, and children with MUAC measurements <12.5 were considered malnourished [14].

The data was analysed using SPSS version 22 (IBM Corp.), and graphs were plotted with GraphPad Prism version 8.4.2 (GraphPad Software, Inc.). The data was summarised using descriptive statistics. Continuous data is presented as mean ± standard deviation (SD), and the categorical data is presented as absolute numbers and percentages. At individual level all Griffiths subscale scores for each domain were assessed and a cut-off score of < 50 for the domain’s development quotient (DQ) was applied to identify developmental challenges within each domain [12]. For the population analyses, in line with standard practise [19], a population reference DQ score of 50–99 was considered low to low average (i.e. poor) and score of ≥100 is considered average to high, indicating typical development for the chronological age (i.e good).

For all statistical analyses, data was checked for the assumptions of parametric tests. Thus, the data was checked for normality, using frequency distribution plots, in addition to the Shapiro Wilkes normality test to determine if parametric or non-parametric statistics were to be used. To determine differences between continuous data (between two groups or between one group and a reference mean), the t-test or the Wilcoxon Sign test (non-parametric) was used. To determine differences in categorical variables between two groups, the Fisher’s exact test was used. For all analyses, approximate 95% confidence intervals (CI) were calculated using the modified Wald method [20], and p-values <0.05 were considered significant. For univariate analyses comparing DQ subscale scores between groups, it was hypothesised a priorly that schistosome-infected/stunted/malnourished individuals will have poorer scores than uninfected/healthy individuals, hence statistical tests were one-tailed.

The risk of associated poor cognitive performance scores in relation to *S*. *haematobium* infection or to stunting and malnutrition was calculated using prevalence ratios (PR). The PR was calculated as a ratio of the proportion of infected/stunted/malnourished individuals with the associated poor DQ scores (e.g. for General Development) to the proportion of uninfected/healthy individuals with the same poor DQ scores (e.g. for General Development). A subscale indicator with a PR >1, suggested an increased risk of the associated poor DQ scores from schistosome infection/stunting/malnutrition [21]. The method of attributable fraction (AF) was then used to estimate the proportion of poor DQ scores for each subscale that could be attributed to *S*. *haematobium* infection/stunting/malnutrition. The population attributable fraction (AFp) and attributable fraction in the exposed (AFe) were used to estimate the attributable fractions in the total study population and among the exposed (i.e. schistosome-infected/stunted/malnourished children) respectively, according to Miettinen’s formula [22].

The AF in the exposed population was calculated as:

$$AFe=\frac{\left(RR-1\right)}{RR}$$ (1)

The AF in the total population was calculated as:

AFp=Pe×AFe (2)

Where RR is the risk ratio of poor DQ scores associated with infection/stunting/malnutrition, and Pe is the prevalence of poor DQ scores among the infected/stunted/malnourished population. As all AFs were estimated at the cross-sectional level (i.e. at the baseline survey), the RR was substituted with the PR [21] and AFs were only estimated for morbidity indicators with PR >1.

## Results

### Population and survey characteristics

Out of a total of 166 children recruited into the study aged 6–72 months, 11 (6.6%) were excluded from the study based on low DQ scores (i.e. DQ scores below 50) as well as observations/interactions by/with the psychologists. These children were referred for further clinical attention. Suspected diagnosis of these 11 children included Anxiety, Attention Deficient Disorder (ADD), Attention Deficient Hyperactivity Disorder (ADDH), Seizure Disorder and Intellectual Disabilities (ID).

Of the155 children included in the final analysis, 83 (53.5%) were male and 72 female (46.4%). Age range was 9–72 months (mean± SD = 45.5 ± 15.4 months; 95% CI = 43–48). Anthropometric measures were taken at baseline, from which standardised indices of malnutrition and stunting were determined; mean weight 14.8 ± 3.0 kg (95% CI = 14.3–15.3), mean height 95.7 ± 11.3 cm (95% CI = 93.8–97.6) and mean MUAC 15.8 ± 1.3 cm (95% CI = 15.6–16.0). Prevalence of stunting based on HAZ was 26.8% (37/138; 95% CI = 20.1–34.8), and malnutrition as measured by different indices were 0% (0/139) based on MUAC, 6.6% (9/137; 95% CI = 3.5–12) based on WAZ, and 3.6% based on BAZ.

Parasitology data was available for 141/155 (91%) children; *S*. *haematobium* infection prevalence was 39.7% (56/141; 95% CI = 32.0–48.0) and overall mean infection intensity was 5.4 eggs/10 ml urine (SEM = 1.6; 95% CI = 2.3–8.6). Follow up rates at subsequent surveys were 122/155 (78.7%) at 6 months (survey S1) and 61/122 (50%) at 12 months (survey S2). *S*. *haematobium* prevalence was 8.3% (8/96; 4.1–15.8) at survey S1, and 8.9% (5/56; 3.5–19.7) at survey S2.

### Cognitive performance at baseline

Table 1 summarises the cognitive performance assessment of all children at baseline, based on the Developmental Quotient (DQ) for all six subscales. Using the population reference cut off DQ = 100, the mean DQ for the subscales (i.e. Eye and Hand Coordination EHC, PSE, GM,) and General Development (GD)) were good and so was the GD quotient. The mean population DQ for the FL and LC subscales were poor (i.e. <100) with majority of children (>50%) falling below the reference cut off.

**Table 1:** Summary baseline cognitive assessment based on the developmental quotient.

Subscale	Mean developmental age (SD; 95% CI)	Mean developmental quotient (SD; 95% CI)	Good score n (%)	Poor score n (%)
Foundations of Learning (FL)	43.5 (20.0; 40.3–46.7)	96.25 (21.6; 91.82–98.7)	68 (43.9)	87 (56.1)
Language and Communication (LC)	45.1 (20.0; 41.9–48.3)	96.9 (19.0; 93.8–99.9)	69 (44.5)	86 (55.5)
Eye and Hand Coordination (EHC)	46.7 (19.2; 43.6–49.7)	102.7 (15.9; 100.1–105.2)	96 (61.9)	59 (38.1)
Personal Social and Emotional (PSE)	49.4 (17.9; 46.5–52.2)	110.6 (14.7; 108.3–112.9)	125 (80.6)	30 (19.4)
Gross Motor (GM)	50.9 (18.7; 48.0–53.9)	120.3 (99.4; 104.5–136.0)	131 (84.5)	24 (15.5)
General Development (GD)	47.5 (21.5; 44.1–50.9)	104.0 (17.5; 101.3–106.8)	91 (58.7)	64 (41.3)

Children were classified based on developmental quotient into good (equal to, or above average) and poor scores (below average) using a population reference cut off ≥100 [19].

**Fig 1:**
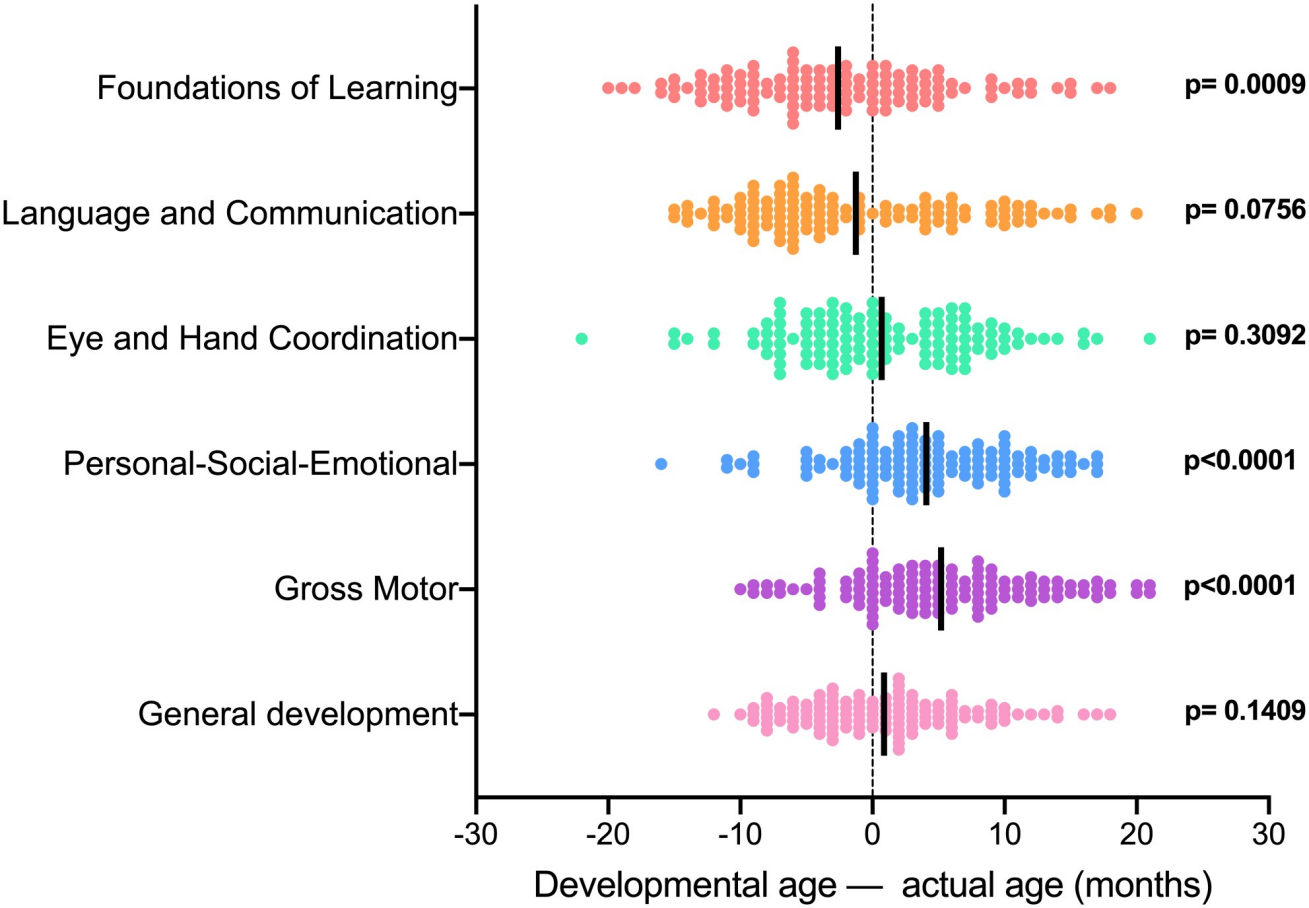
Difference between the developmental and chronological/actual ages for the six subscales. Dotted line represents children performing as expected for their chronological age as per Griffiths test, i.e. the difference between the chronical and developmental age performance. P-values indicate statistical tests comparing the difference between the mean developmental age as indicated by the Griffiths test performance and the mean chronological age. Solid lines = mean.

The developmental age derived based on the Griffiths assessment was compared to the chronological age (actual ages) of children to determine how their normal physical and mental developments deviate or parallel to normal development milestones (Fig 1). Results showed that the children’s developmental age was lower than expected for the FL subscale (p <0.0009), and higher for the PSE (p <0.0001) and GM (p <0.0001) subscales. Developmental versus chronological age for the LC (p = 0.0756) and EHC (p = 0.3092) subscales were comparable.

### *S*. *haematobium* infection and cognitive performance

**Fig 2:**
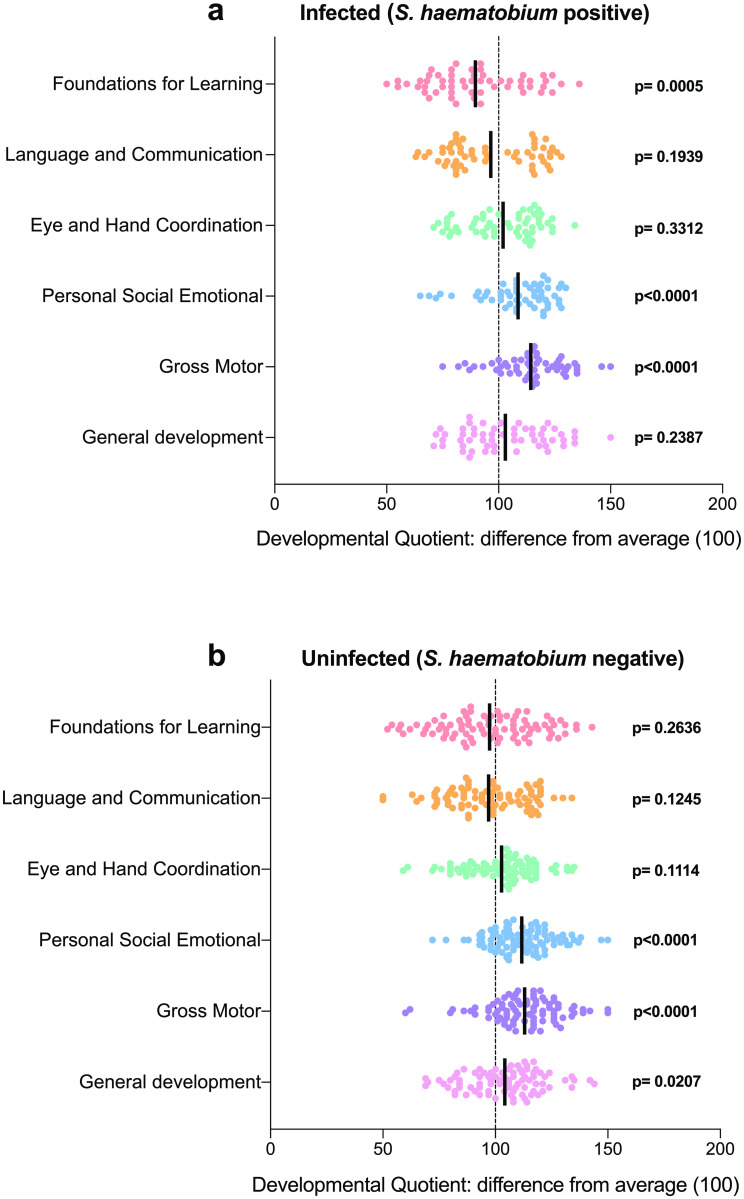
Scatterplot comparing the mean of developmental quotient (DQ) scores to the expected mean reference average for the six subscales. Graph shows baseline DQ scores for a) children positive for *S*. *haematobium* infection, and b) children negative for *S*. *haematobium* infection. Dotted lines represent the mean reference average for DQ (100) [19]. p-values indicate statistical tests for the scores of the children compared to the reference mean of 100. Solid lines = mean.

To determine if any relationship exists between schistosome infection and poor cognitive performance, children were partitioned based on *S*. *haematobium* infection status and their cognitive performance scores. As shown in Fig 2, children positive for *S*. *haematobium* infection had poorer cognitive performance scores for the FL subscale (p = 0.0005), but this was not the case for uninfected children (p = 0.2636). Higher scores were recorded for the GD subscale in the uninfected children, and higher scores recorded for the PSE (p <0.0001) and GM (p <0.0001) subscales for both infected and uninfected children. In both groups, subscales for LC and EHC were comparable to the reference mean (p>0.05).

Table 2 compares cognitive performance of children at baseline, stratified by *S*. *haematobium* infection status. Schistosome infection was significantly associated with FL (p = 0.0068), and this association was true for both younger (9–36 months; p = 0.0305) and older (37–72 months; p = 0.0365) children. There was a significant association between schistosome infection and GD amongst the older group of children (37–72 months; p = 0.0476).

**Table 2:** Cognitive performance classification based on schistosome infection status.

Age category (months)	Subscale classification	*S*. *h*. positive	*S*. *h*. negative	p-value
	**Foundations of Learning**			
All (9–72)	Below for age	41 (70.7%)	44 (48.9%)	**0.0068**
	Normal for age	17 (29.3%)	46 (51.1%)	
9–36	Below for age	9 (90%)	17 (51.5%)	**0.0305**
	Normal for age	1 (10%)	16 (48.5%)	
37–72	Below for age	32 (66.7%)	27 (47.4%)	**0.0365**
	Normal for age	16 (33.3)	30 (52.6%)	
	**Language and Communication**			
All (9–72)	Below for age	32 (55.2%)	52 (57.8%)	0.4428
	Normal for age	26 (44.8%)	38 (42.2%)	
9–36	Below for age	9 (90%)	27 (69.7%)	0.4763
	Normal for age	1 (10%)	6 (30.3%)	
37–72	Below for age	23 (47.9%)	25 (43.9%)	0.4132
	Normal for age	25 (52.1%)	32 (56.1%)	
	**Eye and Hand Coordination**			
All (9–72)	Below for age	26 (44.8%)	32 (35.6%)	0.1696
	Normal for age	32 (55.2%)	58 (64.4%)	
9–36	Below for age	8 (80.0%)	18 (54.5%)	0.1413
	Normal for age	2 (20.0%)	15 (45.5%)	
37–72	Below for age	18 (37.5%)	14 (24.6%)	0.1109
	Normal for age	30 (62.5%)	43 (75.4%)	
	**Personal-Social and Emotional**			
All (9–72)	Below for age	12 (20.7%)	16 (17.8%)	0.4069
	Normal for age	46 (79.3%)	74 (82.2%)	
9–36	Below for age	4 (40.0%)	11 (33.3%)	0.4879
	Normal for age	6 (60.0%)	22 (66.7%)	
37–72	Below for age	8 (16.7%)	5 (8.8%)	0.1772
	Normal for age	40 (83.3%)	52 (91.2%)	
	**Gross Motor**			
All (9–72)	Below for age	10 (17.2%)	14 (15.6%)	0.4783
	Normal for age	48 (82.8%)	76 (84.4%)	
9–36	Below for age	4 (40.0%)	7 (21.2%)	0.2141
	Normal for age	6 (60.0%)	26 (78.8%)	
37–72	Below for age	6 (12.5%)	7 (12.3%)	0.6010
	Normal for age	42 (87.5%)	50 (87.7%)	
	**General development**			
All (9–72)	Below for age	29 (50.0%)	33 (36.7%)	0.0759
	Normal for age	29 (50.0%)	57 (63.3%)	
9–36	Below for age	8 (80.0%)	18 (54.5%)	0.1413
	Normal for age	2 (20.0%)	15 (45.5%)	
37–72	Below for age	21 (43.8%)	15 (26.3%)	**0.0476**
	Normal for age	27 (56.2%)	42 (73.7%)	

Data are presented as n (%), and p-values indicate Fishers exact tests for indices. S.h., S haematobium

### Poor cognitive performance attributable to *S*. *haematobium* infection

**Fig 3:**
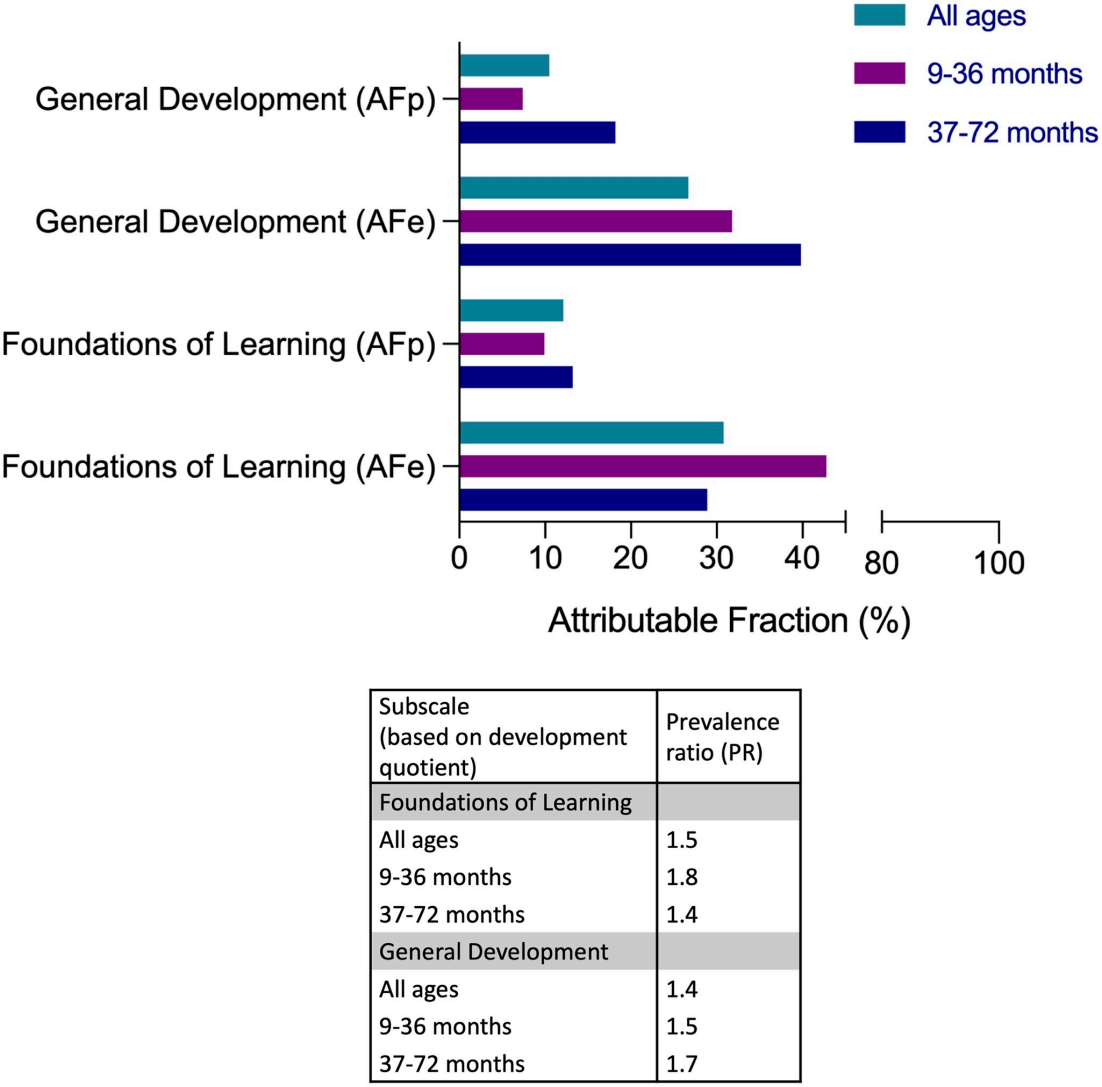
Estimated proportion of poor cognitive performance (based on development quotient) attributable to *S*. *haematobium* infection. AFe = Attributable fraction in the infected population, AFp = attributable fraction in the total population. Attributable fractions were estimated for indices with PR >1.

Since cognitive performance scores in children may relate to different factors inherent to specific populations, we determined how much of poor cognitive performance was attributable to schistosome infection, focusing on the subscales that showed a significant association with *S*. *haematobium* infection (i.e. FL and GD subscales). As shown in Fig 3, 30.8% (AFe) of poor FL was attributable to schistosome infection in the infected population, and in the total population (AFp), 12.1%. When children were grouped into age categories, the proportion of poor FL attributable to schistosome infection was higher for children ≤36 months (AFe = 42.8%) and 28.9% for children >36 months. Similarly, poor GD was attributable to schistosome infection in the infected population (AFe = 26.7%) and in the total population but lower (AFp = 10.5%). The proportion of poor GD attributable to schistosome infection was higher for children >36 months (AFe = 40%) and was 32% for children ≤36 months.

### Effects of praziquantel treatment on cognitive performance

To determine the effect of treatment on cognitive performance, a subset of children were followed up. These were children with full ECD assessments from the baseline survey as well as full *S*. *haematobium* parasitology data. This gave a total of 79 children, made up of 29 children who had been positive for *S*. *haematobium* infection at baseline and had been successfully treated for the infected as confirmed at by the treatment efficacy check and 50 children who had been free of schistosome infection at baseline. Due to the heterogeneity in the children’s ages and sexes, statistical analyses were conducted for infected and uninfected children relative to the reference data for their age groups.

**Fig 4:**
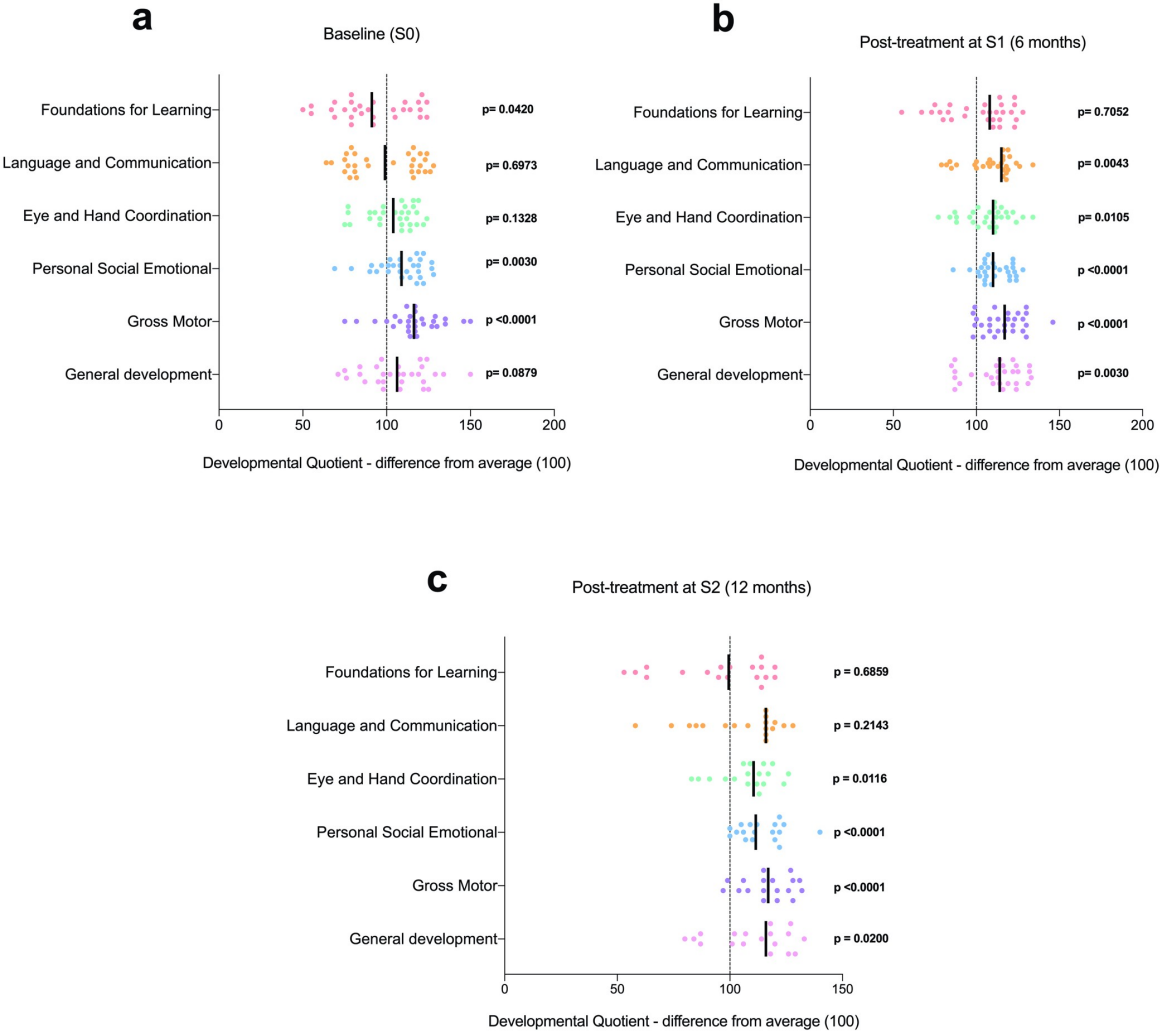
Six- and 12-month time course of developmental quotient (DQ) scores for treated *S*. *haematobium* positive children. Scatterplots show DQ scores for a) children positive and treated for *S*. *haematobium* infection at baseline (n = 29), b) children present at S1 survey (6 months later) and tested negative for *S*. *haematobium* infection (n = 29), and c) children present at S2 survey (12 months later) who remained negative for *S*. *haematobium* infection (n = 18). Dotted lines represent the mean reference average for DQ (100). p-values indicate statistical tests for either a one sample t-test or Wilcoxon sign test (based on normality) comparing to the hypothetical mean of 100. Solid lines = mean.

Thus the cognitive performance subscale scores (based on DQ) for the schistosome-positive children at baseline who were treated, confirmed negative at post-treatment follow-up, were compared to the normal population reference mean (≥100) (Fig 4). The sample size at S1(6 months) was 29. At survey S2 (12 months), a few children from this subset were lost to follow up or were reinfected (n = 2), with data for 18 individuals available for analysis. Compared to the population reference mean, subscales FFL and LC were below normal at baseline (pre-treatment), although the difference was significant only for FL (p = 0.0420) (Fig 4a). Nonetheless, these improved at 6 months post-treatment so that the mean vales for FL were just above the normal level of 100 although not significant (p = 0.7052) while LC mean values were significantly higher that the population reference of 100 (p = 0.0043) (Fig 4b). EHC (p = 0.0105), PSE (p <0.0001), GM (p <0.0001), and GD (p = 0.0030) were all significantly higher than the normal population reference (Fig 4b). Similarly, scores for all six subscales were near or above normal at 12 months post-treatment within the same subset of children (Fig 4c).

**Fig 5:**
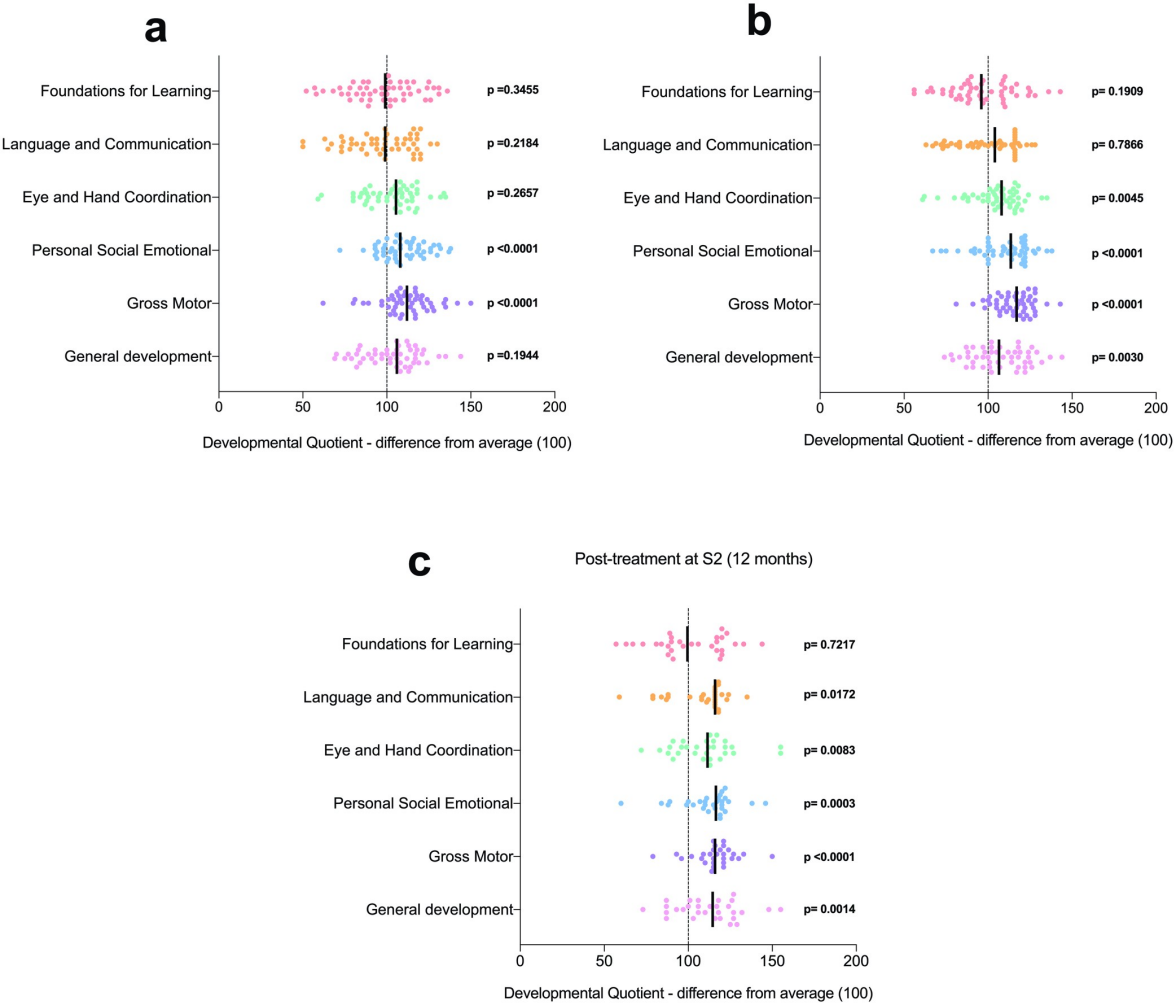
Six- and 12-month time course of developmental quotient (DQ) scores for untreated/uninfected children. Scatterplots show DQ scores for a) children negative for *S*. *haematobium* infection at baseline (n = 50), b) children present at S1 survey (6 months later) and remained negative for *S*. *haematobium* infection (n = 50), and c) children present at S2 survey (12 months later) who remained negative for *S*. *haematobium* infection (n = 29). Dotted lines represent the mean reference average for DQ (100). p-values indicate statistical tests for either a one sample t-test or Wilcoxon sign test (based on normality) comparing to the hypothetical mean of 100. Solid lines = mean.

The analysis was repeated for a subset of children who were uninfected at baseline and remained uninfected (confirmed by egg count) through from surveys S1 (6 months) to S2 (12 months) (n = 50). Similarly, there were some lost to follow up for this subset of children at survey S2, with data for 29 individuals available for analysis. Compared to baseline subscale scores for schistosome-positive children (see Fig 4a), all six subscale scores were near or above normal for uninfected children and this was similar for scores at 6 and 12 months. However, for both infected/treated and uninfected children, subscale scores for PSE and GM were significantly higher than the expected population reference mean at all three time points (see Figs 4 and 5).

### Poor cognitive performance attributable to stunting

We went on to determine the impact of growth and nutritional status on the development measures among the children. As shown in Table 3, development scores showed that stunting was significantly associated with EHC (p = 0.0042), GM (p = 0.0099), and GD (p = 0.0014) subscale DQ scores. When grouped according to age category, stunting was significantly associated with DQ scores for LC (p = 0.0351) for younger children (≤36 months) and with GD (p = 0.0014) in older children (>36 months). Association between all six subscale DQ scores and malnutrition as determined by HAZ and BAZ were not significant (p>0.05).

**Table 3:** Cognitive performance classification based on stunting status.

Age category (months)	Subscale Classification	Stunted	Normal	P value
	**Foundations of Learning**			
All (9–72)	Below for age	25 (67.6%)	53 (52.5%)	0.0814
	Normal for age	12 (32.4%)	48 (47.5%)	
9–36	Below for age	10 (62.5%)	15 (62.5%)	0.6282
	Normal for age	6 (37.5%)	9 (37.5%)	
37–72	Below for age	15 (71.4%)	38 (49.4%)	0.0589
	Normal for age	6 (28.6%)	39 (50.6%)	
	**Language and Communication**			
All (9–72)	Below for age	25 (67.6%)	54 (53.5%)	0.0980
	Normal for age	12 (32.4%)	47 (46.5%)	
9–36	Below for age	16 (100%)	18 (75.0%)	**0.0351**
	Normal for age	0 (0.0%)	6 (25.0%)	
37–72	Below for age	9 (42.9%)	36 (46.8%)	0.4735
	Normal for age	12 (57.1%)	41 (53.2%)	
	**Eye and Hand Coordination**			
All (9–72)	Below for age	22 (59.5%)	33 (32.7%)	**0.0042**
	Normal for age	15 (40.5%)	68 (67.3%)	
9–36	Below for age	12 (75.0%)	12 (50.0%)	0.1046
	Normal for age	4 (25.0%)	1250.0%)	
37–72	Below for age	10 (47.6%)	21 (27.3%)	0.0674
	Normal for age	11 (52.4%)	56 (72.7%)	
	**Personal-Social and Emotional**			
All (9–72)	Below for age	11 (29.7%)	16 (15.8%)	0.0601
	Normal for age	26 (70.3%)	85 (84.2%)	
9–36	Below for age	8 (50.0%)	7 (29.2%)	0.1587
	Normal for age	8 (50.0%)	17 (70.8%)	
37–72	Below for age	3 (14.3%)	9 (11.7%)	0.4984
	Normal for age	18 (85.7%)	68 (88.3%)	
	**Gross Motor**			
All (9–72)	Below for age	11 (29.7%)	11 (10.9%)	**0.0099**
	Normal for age	26 (70.3%)	90 (89.1%)	
9–36	Below for age	6 (37.5%)	4 (16.7%)	0.1322
	Normal for age	10 (62.5%)	20 (83.3%)	
37–72	Below for age	5 (23.8%)	7 (9.1%)	0.0793
	Normal for age	16 (76.2%)	70 (90.9%)	
	**General Development**			
All (9–72)	Below for age	24 (64.9%)	35 (34.7%)	**0.0014**
	Normal for age	13 (35.1%)	66 (65.3%)	
9–36	Below for age	12 (75.0%)	12 (50.0%)	0.1046
	Normal for age	4 (25.0%)	12 (50.0%)	
37–72	Below for age	12 (57.1%)	23 (29.9%)	**0.0212**
	Normal for age	9 (42.9%)	54 (70.1%)	

Data are presented as n (%), and p-values indicate Fishers exact tests for indices.

**Fig 6:**
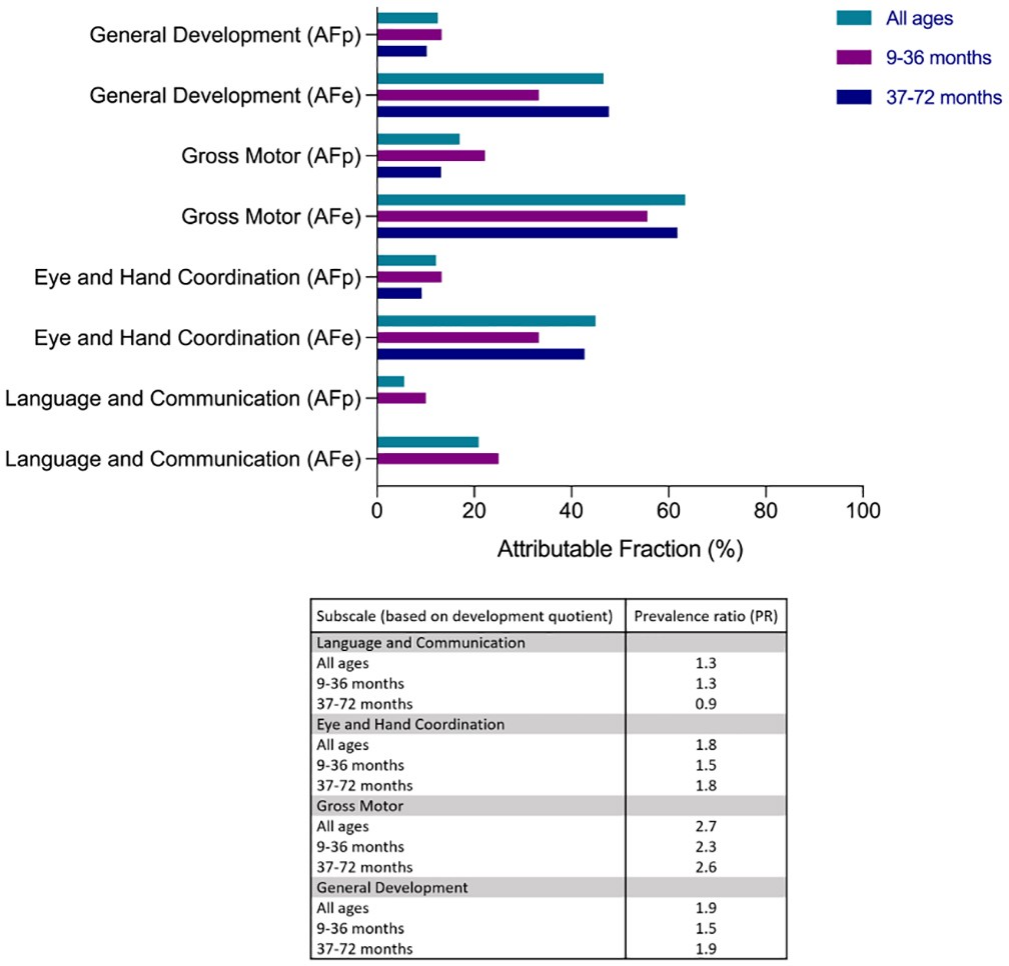
Estimated proportion of poor cognitive performance (based on development quotient) attributable to stunting. AFe = Attributable fraction in the stunted population, AFp = attributable fraction in the total population. Attributable fractions were estimated for indices with PR >1.

Based on the initial analysis, we then determined how much of poor development scores were attributable to stunting (Fig 6). Among the stunted population, poor scores for the four subscales were highly attributable to stunting (AFe: GM = 63.4%, GD = 46.6%, EHC = 45%, and LC = 21%). When children were grouped according to age category, the proportion of poor scores attributable to stunting among older children (i.e. >36 months) was higher (AFe: GM = 61.8%, GD = 47.7%, and EHC = 42.7%) than that in younger children (i.e. ≤36 months; AFe: GM = 55.6%, GD = 33.3%, and EHC = 33.3%). In the total population, poor subscale scores were attributable to stunting although lower (AFp: GM = 17.0%, GD = 12.5%, EHC = 12.1%, and LC = 5.6%).

## Discussion

Indexes monitoring early child development such as The Sustainable Child Development Index [23] and Early Child Development Index, (https://www.unicef.org/socialpolicy/files/child-development-index.pdf) have indicated that children in sub-Saharan Africa are doing worse than those from any other region in terms of reaching developmental milestones. However, there is a paucity of comprehensive child development data from African children, particularly those in rural areas to fully inform such indices and to identify locally relevant interventions. Therefore, we conducted a comprehensive assessment of child development at community level to provide baseline characterisation of child development patterns in rural Zimbabwe.

Our analysis considered development in children aged 6 to 72 months in five domains: Eye and Hand Coordination (EHC), Personal-Social-Emotional (PSE), Language and Communication (LC), Foundations of Learning (FL) and Gross Motor (GM). We also combined the scores from these domains to calculate a score for General Development as per protocol) [12]. Our study detected that 6.6% of the children faced developmental or neurological challenges/disorders that warranted further clinical attention. Suspected diagnosis among these children included Anxiety, Attention Deficient Disorder (ADD), Attention Deficient Hyperactivity Disorder (ADDH), seizure disorder and intellectual disabilities (ID). Our study sample size meant that informative comparisons on these suspected disorders could not be made with findings other African communities. Nonetheless, studies in Africa focusing on single developmental challenges have indicated higher prevalences of such disorders. For example, a study in rural Uganda [24] reported up to 25% prevalence of anxiety disorders in children less than 5 years old. Separate studies have reported lower prevalences of ADHD e.g. 1.5% in the general population in Nigeria [25] but higher prevalences e.g. 7% of children aged below 10 years in paediatric neurology and psychiatry clinics in Uganda reported [26]. Children within communities especially rural communities, are not routinely screened for cognitive or neurodevelopmental development disorders and subtle indicators that can be detected by comprehensive child development assessment are not detected in the normal baby growth monitoring activities (see e.g. [27]). Our identification of the 11 children requiring further clinical investigation and intervention indicates a need for more studies and screening programs to document and diagnose these disorders that are going untreated in Africa children [28].

Our findings that the Zimbabwean children performed better than average in the GM domain are consistent with finding from other studies in Africa. A study in South African children compared gross motor skills in 3–5 year old children from rural low-income backgrounds to those from urban backgrounds (both low and high income) [29]. In our study, just over half of the children performed as expected for their age or better for the overall general development score (GD). This is lower that what has been reported in the South African study where the overall majority of the children (93%) performed well for their age being classified as having average or better scores. This was also the case in Nigerian children [30]. Interestingly, in the South African study, rural children performed better that urban children [29] although the reasons for this finding were not explored. In the Zimbabwean study, it is likely that the GD score was brought down by poor performances in some of the subscales. The Griffiths Developmental Scales compare a child’s chronological age to a developmental age derived from his/her performance on test items in the subscales. An analysis of this cohort’s performance showed their developmental level as lower than the chronological age in the FL domain, but higher for PSE and GM. Developmental ages for LC and EHC were comparable.

There is a paucity of studies in children aged below 5 years characterising the PSE development of children independent of confounders such as infection and non-infectious conditions. Nonetheless, a large, study comparing the performance of 7 year olds from the Millennium Cohort [31] investigating ethnic differences in children’s socioemotional difficulties indicated that after accounting for maternal and family environment factors Black African children had significantly fewer socioemotional difficulties. This is consistent with our finding that the majority of children in our study performed well (or above expectation) for their age in the PSE domain.

The cognitive FL assesses skills and abilities essential for early childhood learning that set the pace and trajectory for future educational achievement. FL measures critical psychometric constructs including cognitive skills for learning, executive function, ways of thinking, problem solving, organizing and information planning, analytical thought, memory and play [32]. Data from this study shows children’s lowest performance was in this domain. The FL domain would be improved by cognitive simulation tools and activities. According to the UNICEF Country Profiles for Early Childhood Development report (https://nurturing-care.org/resources/country-profiles/) only 37% of Zimbabwean children received early stimulation at home, 28% were in attendance of early childhood education and only 3% have books in the home. Persistent gaps in children’s development in cognitive and non-cognitive domains have been noted between children from disadvantaged backgrounds and peers from more advantaged backgrounds. Enriching caregiving practices are essential for normal child development. Children with responsive caregivers, and those who are in more stimulating, environments, are reported to be more cognitively advanced at the start of school than children from less stimulating homes. In addition frequent interaction between parent/caregiver through e.g. play, has been reported to promote the children’s cognitive, social and emotional development [33].

A study on the challenges faced by children aged 5 years and below conducted in all the provinces of Zimbabwe [34], showed that the majority of children attending ECD classes came from backgrounds fraught with challenges such as hunger, economic hardships, poor mobility of parents, lack of infrastructure and low parental knowledge about the importance of ECD education. Hunger and poor nutrition contribute to stunting and several studies [35,36] have shown a link between stunting and poor child development in low- and middle-income countries. We detected a 26.8% prevalence of stunting, similar to the prevalence of 26% reported in Zimbabwean children aged 5 years and below in 2018 (http://fnc.org.zw/documents/). In this study, stunting was significantly associated with lower scores for EHC, GM, and GD domains across all age groups. Stunting in the first 5 years of life has both immediate and long-term effects in children [37]. For example, work in Benin has shown that stunting compromises cognitive development in children [35], while a cohort study in 8 Low-Middle Income Countries including those in Africa demonstrated that stunting within the first 6 months which persisted for 60 months was associated with lower cognitive development in children at 5 years old [36]. UNICEF recognizes malnutrition as having potential adverse consequences on child survival and long-term well-being with far reaching consequences for economic productivity and human capital development. This increases the urgency to accelerate progress towards Sustainable Development Goal 2 to end hunger, achieve food security and improved nutrition, to also attain the other SDGs.

In addition to nutrition, other factors such as childhood infections [38] also affect early child development. We have recently reported that early childhood infections with schistosomes affect metabolism in children aged 5 years and below, [39], which may have a subsequent impact on ECD. Whilst the detrimental effects of helminth infections (both STH [4] and schistosomes [7]) have been previously reported, to date there has been no study investigating the impact of schistosome infection on ECD focusing on children aged 5 years and below. In this study we showed that children infected with schistosomes scored significantly lower in the FL domain across all ages while older children (aged 37–72 months) scored significantly lower in GD. Overall, in the schistosome positive children, 30.8% of the poor FL scores were attributable to schistosome infection. Thus, it is not surprising that 6 months following treatment, these scores increased; and by 12 months post treatment, the scores for schistosome infected children improved across all domains, to be similar to those of previously uninfected children. Smaller sample sizes for the cohort studies mean more detailed subgroup analyses could not be conducted. Nonetheless, our findings are consistent with findings from quantitative studies in older children [6].

In conclusion, we have demonstrated that just over 50% of Zimbabwean children in rural areas are on course for normal child development; with many being more advanced for their age in GM and PSE domains. Nevertheless, the children do face developmental challenges in early childhood, particularly in FL. This reflects several factors including those identified by the WHO, including lack of early stimulation at home, lack of access to early childhood education and lack of books in the home. Poor development scores were also attributable to stunting and schistosome infection, with the impact of the latter being reversed by curative antihelminthic treatment. The children’s low FL scores and the impact of S *haematobium* infection, stunting and malnutrition among other factors should be of concern, as they can be a predictor of future educational and occupational challenges. Taken together, the findings strengthen the call for the treatment of paediatric schistosomiasis, accessibility to cognitive stimulation tools and improved nutrition to improve childhood health outcomes and accelerate progress towards the SDGs.

## Supporting information

S1 Fig Study design. (TIF)
