# Supplementary figures and images for: Assessing early child development and its association with stunting and schistosome infections in rural Zimbabwean children using the Griffiths Scales of Child Development
Source: PLoS Negl Trop Dis. 2021 Aug 11;15(8):e0009660. doi: 10.1371/journal.pntd.0009660 (PMC8357151; doi:10.1371/journal.pntd.0009660)

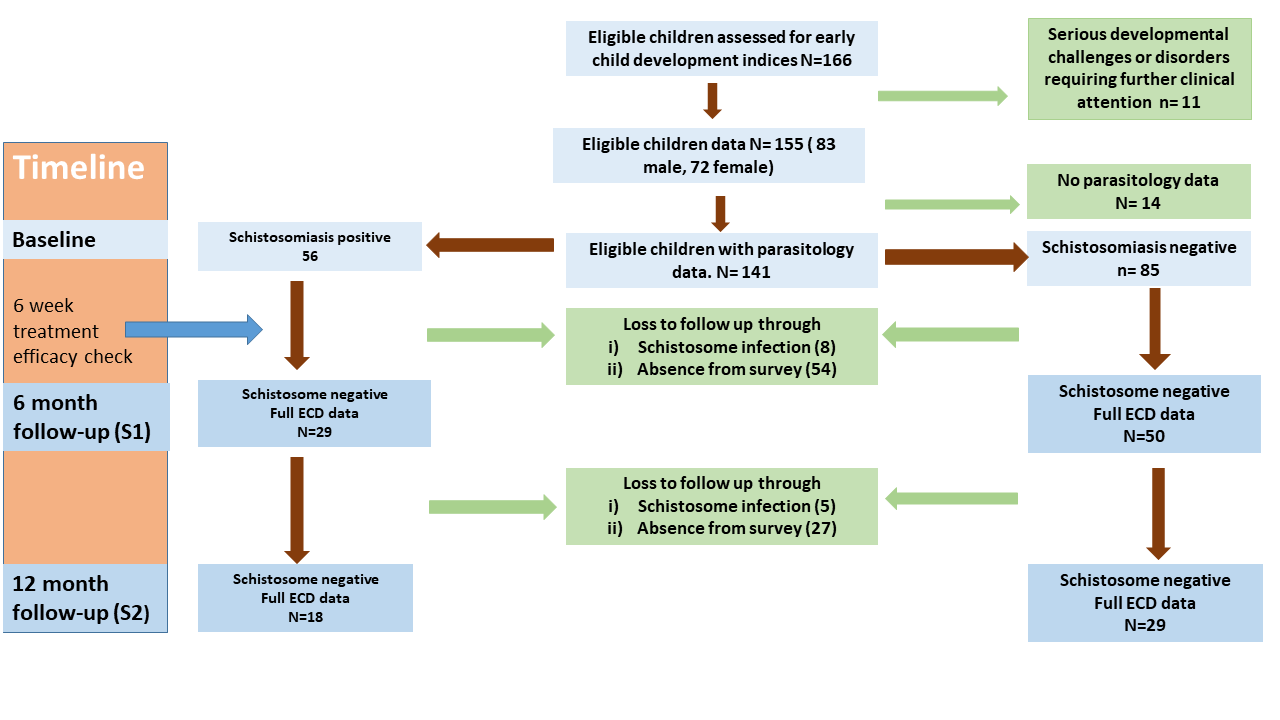

Supplement: S1 Fig — (TIF) [file pntd.0009660.s001.tif]
